# Changes in the blood routine, biochemical indexes and the pro-inflammatory cytokine expressions of peripheral leukocytes in postpartum dairy cows with metritis

**DOI:** 10.1186/s12917-019-1912-y

**Published:** 2019-05-21

**Authors:** Luying Cui, Heng Wang, Yanan Ding, Jun Li, Jianji Li

**Affiliations:** 1 College of Veterinary Medicine, Yangzhou University, 12 East Wenhui Rd, Yangzhou, 225009 Jiangsu China; 2 Jiangsu Co-innovation Center for the Prevention and Control of Important Animal Infectious Disease and Zoonoses, 12 East Wenhui Rd, Yangzhou, 225009 Jiangsu China

**Keywords:** Postpartum period, Metritis, Blood routine, Blood biochemistry, Pro-inflammatory cytokines

## Abstract

**Background:**

The aim of the present study was to clarify the changes in complete blood count, blood biochemistry, and the gene expressions of pro-inflammatory cytokines of peripheral white blood cells in postpartum dairy cows with metritis.

**Results:**

The cows were assigned to the control group (*n* = 28) or the metritis group (*n* = 28), retrospectively. Blood samples were taken 7 days before the estimated parturition (− 7 d), on the day of parturition (0 d), and 7 and 30 d after parturition. There was no difference in blood indexes between the control group and the metritis group at − 7 d. The WBC, granulocytes and monocytes were generally higher at 7 and 30 d in the metritis group than the control. In comparison with the controls, all liver function parameters and triglyceride levels at 0, 7 and 30 d, and the creatinine level at 7 and 30 d were higher in cows with metritis. The concentrations of Ca and P at 0, 7 and 30 d, and of glucose at 0 d were lower for cows in the metritis group compared with cows in the control group. Among these parameters, the WBC at 30 d, the aspartate aminotransferase activity (AST) at 7 d exceeded normal ranges (WBC: 5.0 ~ 16.0 × 10^9^/L; AST: 42.5 ~ 98 U/L), whereas the concentrations of glucose and Ca from 0 to 30 d were below normal ranges (glucose: 2.5 ~ 4.5 mmol/L; Ca: 2.2 ~ 2.5 mmol/L) in the metritis group. The gene expressions of pro-inflammatory cytokines in the metritis group were higher than those in the control group, including the IL-1α at 7d, the IL-1β at − 7, 0 and 7 d, the IL-6 at − 7, 0, 7 and 30 d, the IL-8 at 0, 7 and 30 d, and the TNF-α at 7 and 30 d.

**Conclusion:**

The cows with metritis experienced systemic inflammation for 4 weeks after calving, the impaired hepatic function, and the altered metabolic status with increased triglyceride level and decreased concentrations of glucose, Ca and P.

## Background

Postpartum uterine infection is one of the primary causes of reproductive failure in dairy cows [1]. After calving, the bovine uterus undergo extensive modification to reduce in size, remove cellular debris and restore normal structure [2]. Bacterial contamination of the uterus is common in 90% postpartum dairy cows, and most healthy cows are able to clear the uterine bacteria within the first 2 to 3 weeks. It has been documented that around 40% of cows suffered from metritis and 15% had a persistent endometritis [3]. Metritis could be diagnosed within the first 10 days postpartum, and the endometritis mostly occurs during the second through fourth postpartum week [1, 4].

Postpartum metritis and endometritis in dairy cows share common etiological factors, predispose to one another and largely share common treatment [1]. During peripartum period, cows experience sudden nutritional and endocrine changes, leading to compromised immune function [5, 6]. This lowered immune response predisposes the cows to uterine infection [3, 7]. Reduced functional capacity of neutrophils has been reported in cows with metritis [8] and endometritis [9]. Kim et al. found increased blood leucocytes and tumor necrosis factor α (TNF-α) level, but decreased phagocytic capacity in cows with endometritis [7]. Parallel to the changed immune status, the negative energy balance (NEB) status has been observed in postpartum dairy cows, where the food-intake capacity could not cover the enormous energy and protein required for milk production. The nutritional status of dairy cows is critical to immune cell function [5]. It has been shown that the increased risk of metritis is associated with NEB and metabolic diseases (e.g., ketosis-fatty liver complex) during early lactation [10, 11]. In addition, the synthesis and secretion of colostrum by dairy cows imposes a large drain in Ca in the first days postpartum, which may lead to the sudden decrease of Ca concentration and the insufficient availability of ionized Ca [12, 13]. It has been demonstrated that cows with decreased Ca concentration had increased odds of being diagnosed with metritis [12–14].

The postpartum period is important in the reproductive life of dairy cows because of its influence on future fertility. The general determination of immune and metabolic status and the functions of vital organs during postpartum period could provide references to the diagnosis, treatment and prevention protocols for postpartum metritis. The objective of this study was to compare the hematological changes in healthy dairy cows and the cows with metritis by the measurement of peripheral blood routine and biochemical parameters, and the detection of pro-inflammatory cytokines of peripheral leukocytes at different stages. Since uterine infections most commonly occurred around 7 and 30 d postpartum, we selected 7 and 30 d, as well as the day of parturition (0 d) and 7 days before the estimated parturition (− 7 d) as the observation time points. We hypothesized that the cows diagnosed with metritis present altered parameters indicating the immune and metabolic status, as well as the liver and kidney functions.

## Results

### Blood routine

**Fig. 1:**
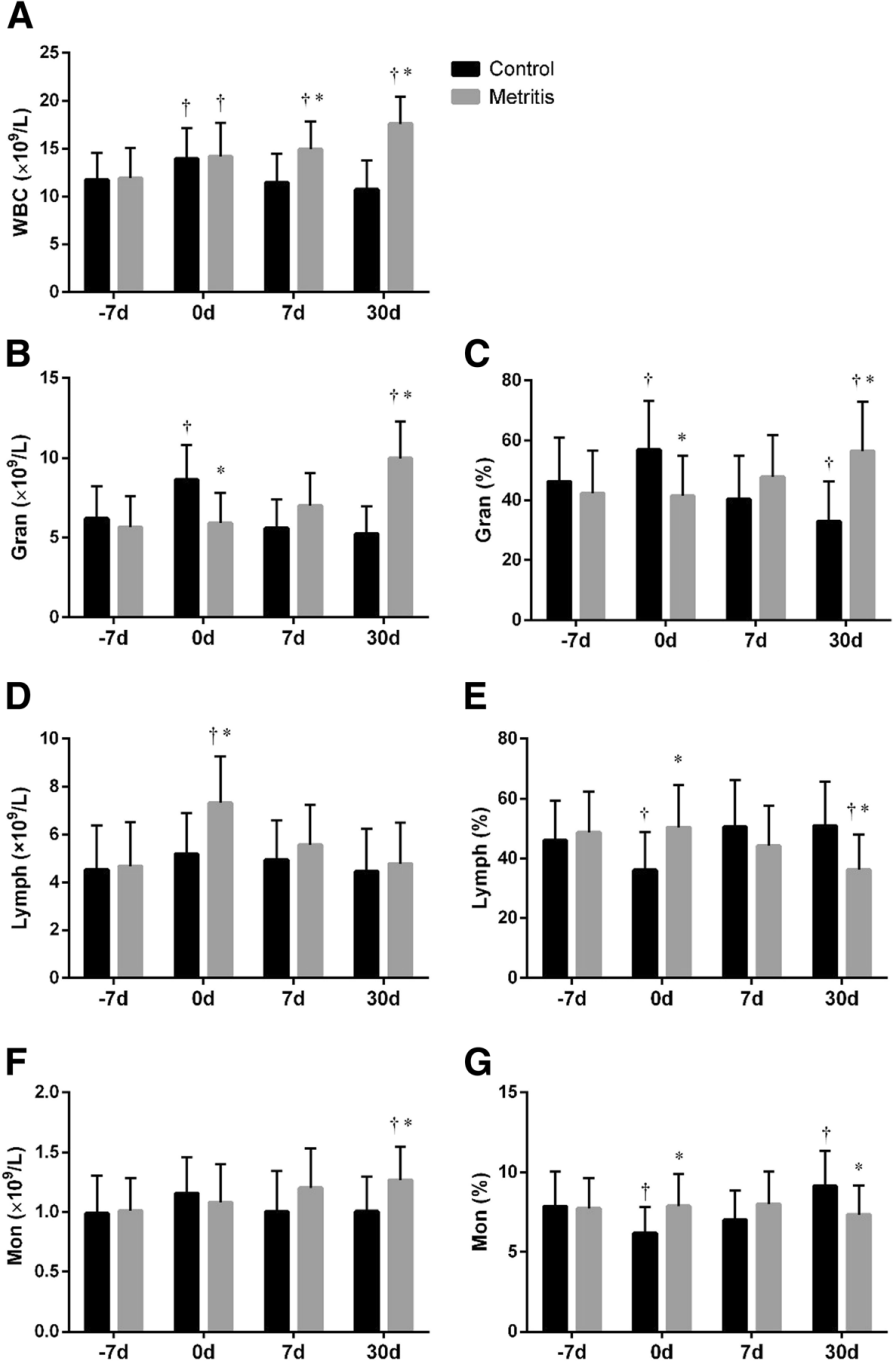
Summed changes (mean ± SD) in (**a**) white blood cell count (WBC), (**b**) Granulocyte count (Gran), (**c**) Granulocyte percentage (Gran%), (**d**) Lymphocyte count (Lymph), (**e**) Lymphocyte percentage (Lymph%), (**f**) Monocyte count (Mon), and (**g**) Monocyte percentage (Mon%) in peripheral blood of cows with metritis (*n* = 28) and control cows (*n* = 28) at 7 days before the estimated parturition (− 7 d), the day of parturition (0 d), and 7 and 30 d postpartum. Asterisks (*) indicates statisticallty significant (*p <* 0.05) difference between the control and the metritis group at the same time point. Crosses (†) indicates statistically significant (*p <* 0.05) difference when compared to − 7 d within the same group

Results of blood routine indicators were shown in Fig. 1 Compared with − 7 d, the WBC in both groups increased (*p <* 0.05) at 0 d (Fig. 1a). Then the WBC of the control group returned to prepartum level (*p* > 0.05), whereas the WBC of the metritis group further increased (*p <* 0.05). The WBC of the metritis group at 30 d (17.6 ± 2.8 × 10^9^/L) exceeded the normal range (5.0 ~ 16.0 × 10^9^/L).

As shown in Fig. 1b and c, both the granulocyte count (Gran, normal range: 2.3 ~ 9.1 × 10^9^/L) and the granulocyte percentage (Gran%, normal range: 30% ~ 65%) in the control group temporally increased (*p* < 0.01) at 0 d. The Gran and Gran% in the metritis group gradually increased from − 7 to 30 d. The Gran at 30 d (10.0 ± 2.3 × 10^9^/L) in the metritis group exceeded the normal range. Compared with the healthy cows, the Gran and Gran% in cows with metritis were lower (*p* < 0.01) at 0 d, but higher (*p* < 0.01) at 30 d.

Compared with the control, both the lymphocyte count (Lymph, normal range: 1.5 ~ 9.0 × 10^9^/L) and the lymphocyte percentage (Lymph%, normal range: 20.0% ~ 60.3%) in the metritis group were higher (*p* < 0.01) at 0 d (Fig. 1d and e). The Lymph% was lower (*p =* 0.01) at 30 d in cows with metritis than that in the healthy cows.

The monocyte count (Mon, normal range: 0.3 ~ 1.6 × 10^9^/L) remained stable in the control group. The Mon in the metritis group was higher (*p* < 0.01) than the control at 30 d (Fig. 1f). The monocyte percentage (Mon%, normal range: 4.0 ~ 12.1 × 10^9^/L) in control group decreased (*p =* 0.01) at 0 d and gradually increased thereafter (Fig. 1g). Compared with healthy cows, the Mon% in cows with metritis were higher (*p =* 0.01) at 0 d, and lower (*p* < 0.01) at 30 d.

### Biochemical indicators

**Fig. 2:**
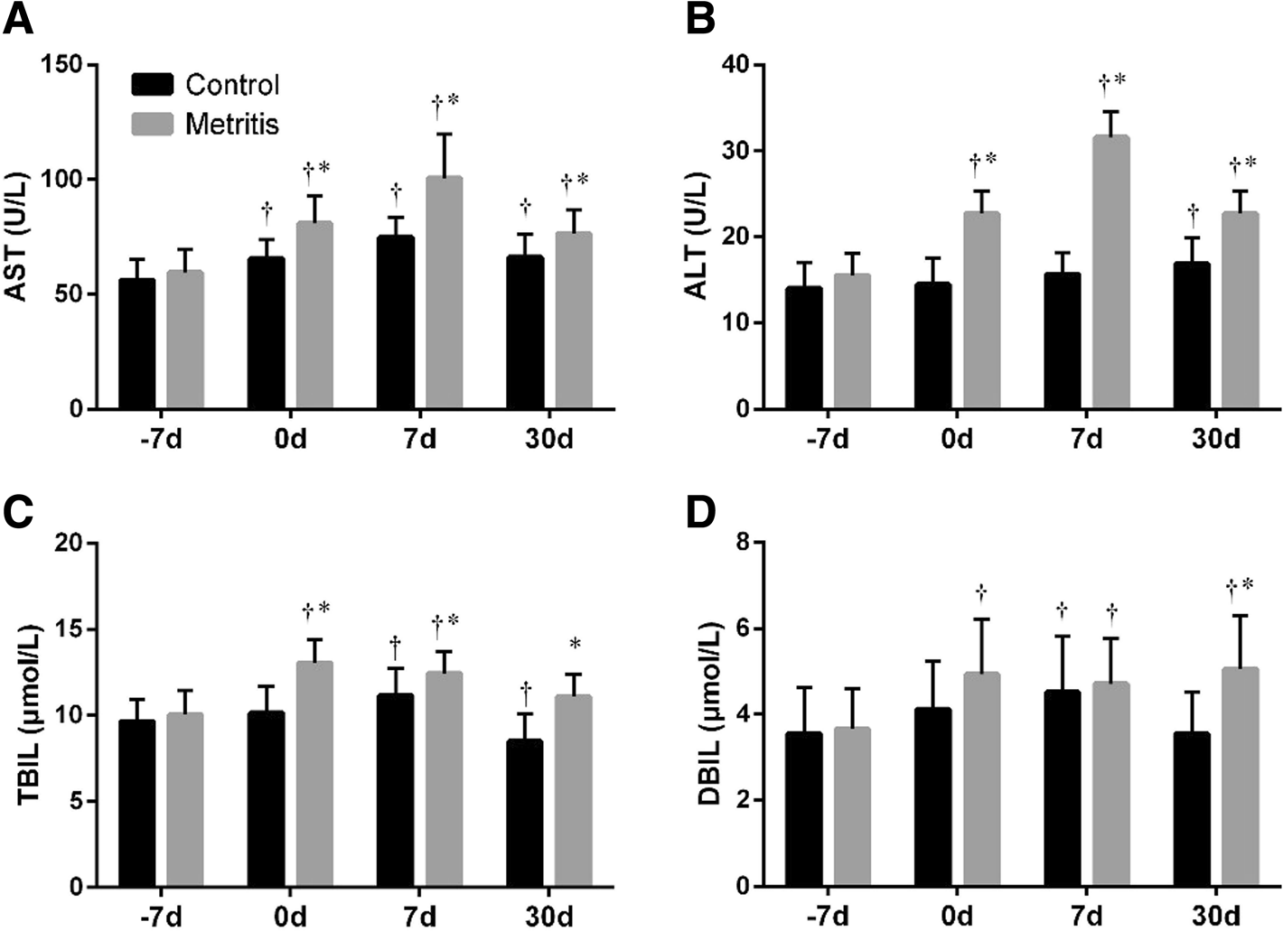
Summed changes (mean ± SD) in (**a**) aspartate aminotransferase (AST) activity, (**b**) alanine aminotransferase (ALT) activity, (**c**) total bilirubin (TBIL) level, and (**d**) direct bilirubin (DBIL) level in serum of cows with metritis (*n* = 28) and control cows (*n* = 28) at 7 days before the estimated parturition (− 7 d), the day of parturition (0 d), and 7 and 30 d postpartum. Asterisks (*) indicates statisticallty significant (*p <* 0.05) difference between the control and the metritis group at the same time point. Crosses (†) indicates statistically significant (*p <* 0.05) difference when compared to − 7 d within the same group

All liver function parameters were within the normal range except aspartate aminotransferase (AST, normal range: 42.5 ~ 98 U/L). Compared with − 7 d, the increased activities of AST and alanine aminotransferase (ALT, normal range: 14 ~ 38 U/L) in the control group were observed from 0 to 30 d, and at 30 d, respectively (Fig. 2a and b). Compared with the control, both the AST and ALT activities of the metritis group were higher (*p <* 0.05) at 0, 7 and 30 d, in which the AST at 7 d (100.7 ± 19.2 U/L) exceeded the normal range. The total bilirubin (TBIL, normal range: 0.2 ~ 17.1 μmol/L) and direct bilirubin (DBIL, normal range: 0.7 ~ 7.5 μmol/L) in the control group increased (*p <* 0.05) at 7 d, and the TBIL decreased (*p =* 0.01) at 30 d compared with − 7 d (Fig. 2c and d). In the metritis group, the TBIL at 0, 7 and 30 d, and the DBIL at 30 d were higher (*p <* 0.05) than those in control group.

**Fig. 3:**
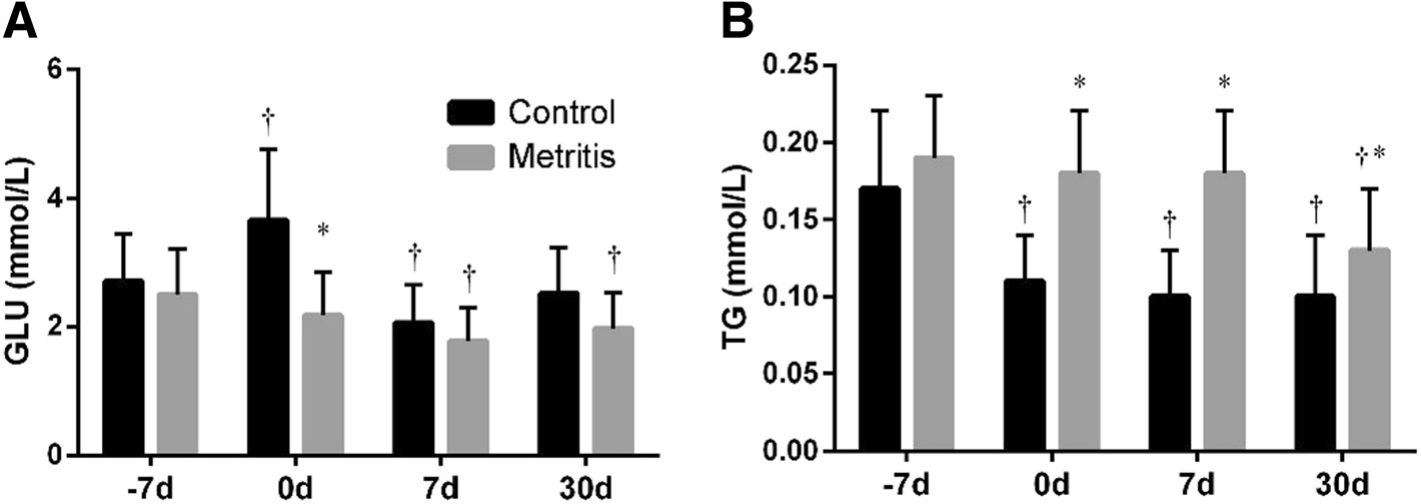
Summed changes (mean ± SD) in (**a**) glucose (GLU) and (**b**) triglyceride (TG) levels in serum of cows with metritis (*n* = 28) and control cows (*n* = 28) at 7 days before the estimated parturition (− 7 d), the day of parturition (0 d), and 7 and 30 d postpartum. Asterisks (*) indicates statisticallty significant (*p <* 0.05) difference between the control and the metritis group at the same time point. Crosses (†) indicates statistically significant (*p <* 0.05) difference when compared to − 7 d within the same group

As shown in Fig. 3a, compared with − 7 d, the glucose increased (*p* < 0.01) at 0 d, decreased (*p =* 0.01) at 7 d, and approximated to the prepartum value at 30 d in the control group. The glucose at 7 d (2.1 ± 0.6 mmol/L) was below the normal range (2.5 ~ 4.5 mmol/L). In the metritis group, the glucose at 0 (2.2 ± 0.7 mmol/L), 7 (1.8 ± 0.5 mmol/L) and 30 d (2.0 ± 0.6 mmol/L) were below the normal range. Compared with − 7 d, the triglyceride (TG) level decreased (all *p <* 0.05) at 0, 7 and 30 d in the control group (Fig. 3b). Compared with the control, the TG levels were higher (*p <* 0.05) at 0, 7 and 30 d in the metritis group.

**Fig. 4:**
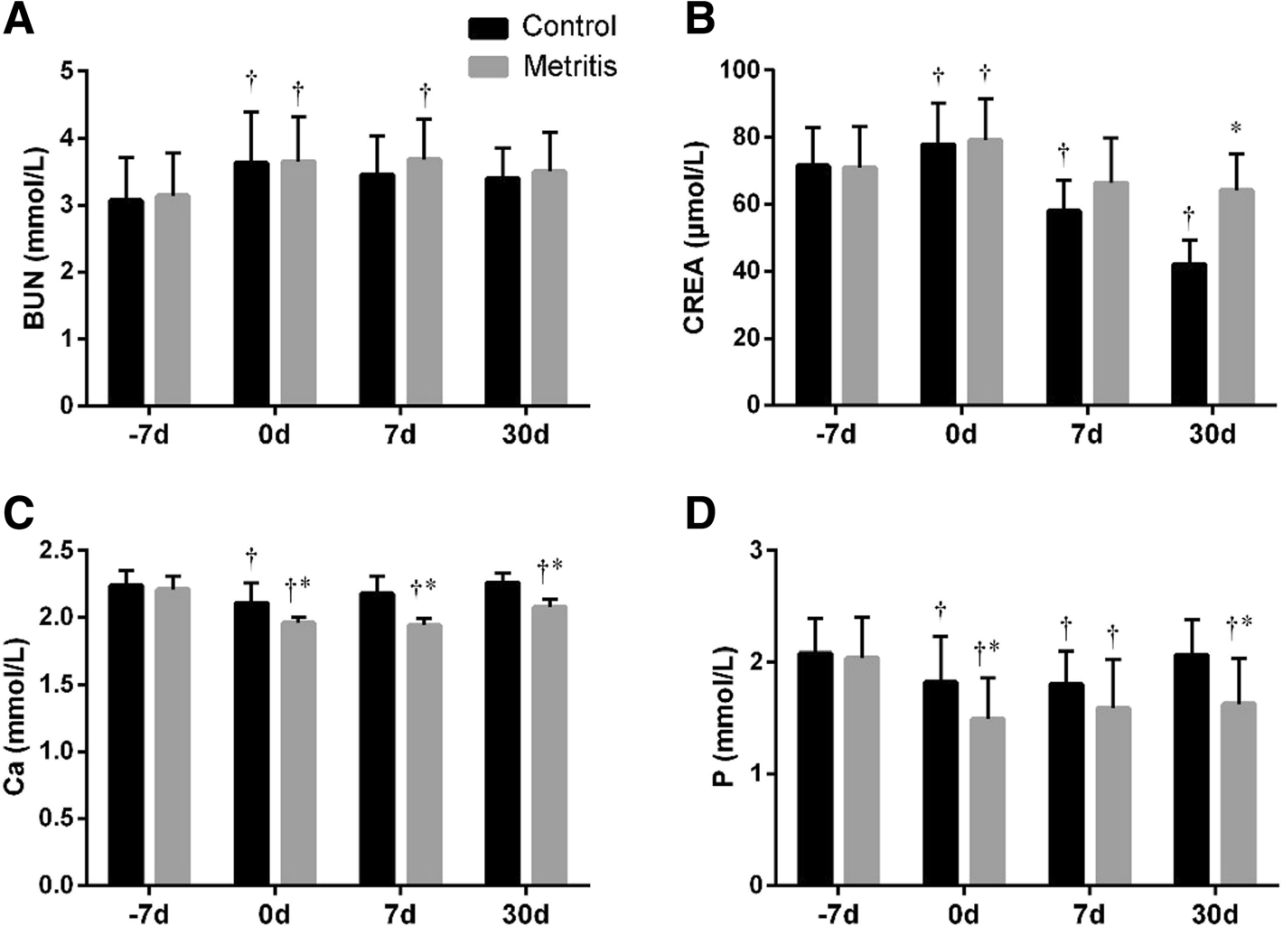
Summed changes (mean ± SD) in (**a**) blood urea nitrogen (BUN) and (**b**) creatinine (CREA) levels, and (**c**) Ca and (**d**) P concentrations in serum of cows with metritis (*n* = 28) and control cows (*n* = 28) at 7 days before the estimated parturition (− 7 d), the day of parturition (0 d), and 7 and 30 d postpartum. Asterisks (*) indicates statisticallty significant (*p <* 0.05) difference between the control and the metritis group at the same time point. Crosses (†) indicates statistically significant (*p <* 0.05) difference when compared to − 7 d within the same group

As shown in Fig. 4a, the blood urea nitrogen (BUN) of both groups showed a temporary increase (*p =* 0.01) at 0 d. No difference was observed in between groups at each time point. The creatinine (CREA) concentration increased (*p =* 0.04) at 0 d, then gradually decreased afterwards (Fig. 4b). The CREA in the metritis group at 30 d were higher (*p* < 0.01) than the control.

Compared with − 7 d, the Ca and P concentrations in the control group decreased (*p <* 0.05) at 0 d, and returned (*p* > 0.05) the prepartum level at 30 d (Fig. 4c and d). Compared with the healthy cows, the Ca and P were lower (*p <* 0.05) at 0 and 30 d, and the Ca at 7 d in cows with metritis. The Ca were below the normal range (2.2 ~ 2.5 mmol/L) at 0 (2.11 ± 0.15 mmol/L; 1.96 ± 0.04 mmol/L) and 7 d (2.18 ± 0.13 mmol/L; 1.94 ± 0.05 mmol/L) in both groups, and at 30 d (2.08 ± 0.06 mmol/L) in the metritis group.

### Pro-inflammatory cytokine gene expressions

**Fig. 5:**
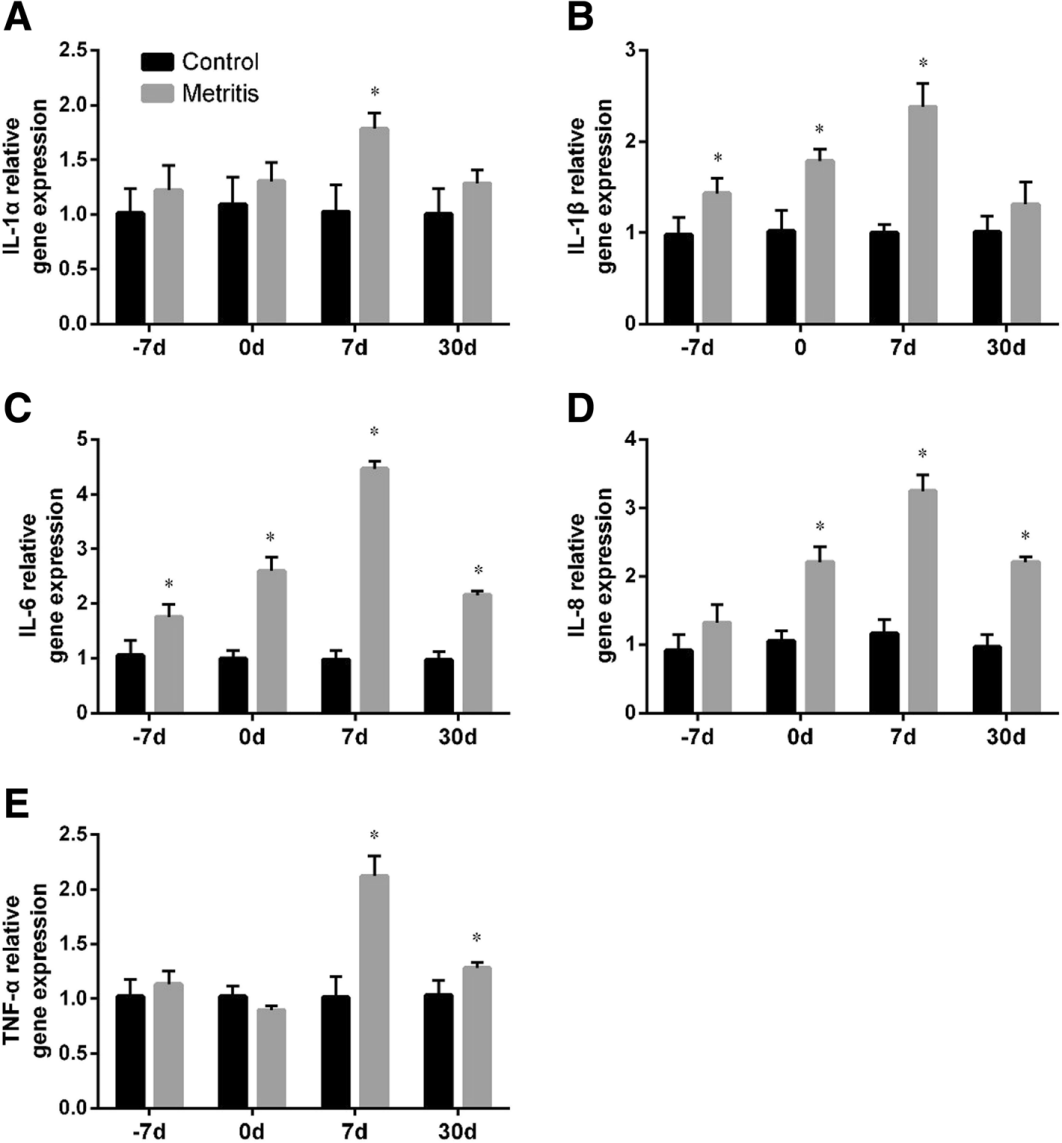
Summed changes (mean ± SD) in the gene expressions of (**a**) IL-1α, (bb) IL-1β, (**c**) IL-6, (**d**) IL-8, and (**e**) TNF-α in peripheral blood of cows with metritis (*n* = 28) and control cows (*n* = 28) at 7 days before the estimated parturition (− 7 d), the day of parturition (0 d), and 7 and 30 d postpartum. Asterisks (*) indicates statisticallty significant (*p <* 0.05) difference between the control and the metritis group at the same time point

Compared with − 7 d, the gene expressions of pro-inflammatory cytokines in healthy cows, including interleukin (IL)-1α, IL-1β, IL-6, IL-8, and TNF-α, increased (all *p <* 0.05) at 0 d, then decreased after parturition. The mRNA expressions of IL-1α and IL-1β at 7 and 30 d approximated to the prepartum level. The gene expressions of IL-6 and IL-8 at 7 and 30 d, and TNF-α at 30 d were lower (all *p <* 0.05) than those at − 7 d. As shown in Fig. 5, compared with the control, the gene expressions of pro-inflammatory cytokines were generally higher in the metritis group.

## Discussion

In the present study, the cows developed metritis presented (1) higher WBC, Gran, and Mon, and the gene expressions of cytokines, (2) higher AST activity and TG level, and lower glucose level, and (3) lower concentrations of Ca and P after calving. In healthy dairy cows, changes in blood parameters and the cytokine gene expressions were mostly obvious at 0 d.

The increases of WBC and Gran in healthy cows at 0 d were probably caused by the stress response at parturition [1]. It has been reported that the leukocyte counts reached the peak level at the day of calving and return to normal values within a few days postpartum [15–17]. The stress pattern was evident in the differential count with an increase of neutrophils [18]. No change was found in Lymph in our result, which was consistent with some of previous reports [19, 20]. Other reports have found decreased lymphocytes at the day of calving [21, 22]. The cows in the metritis group presented lower values of Gran and Gran%, and higher values of Lymph and Lymph% at 0 d in comparison to their healthy counterpart. These differences might indicate the altered immune status of the cows with metritis, which requires further investigations. The WBC, Gran and Mon continued to increase in cows with metritis at 7 and 30 d. Such changes in cows have been reported in a few studies [2, 7, 23, 24], indicating a potential persistent peripheral inflammatory response in the cows with uterine infection.

AST and ALT are the most frequently utilized and specific indicators of hepatocyte injuries [25, 26]. TBIL and DBIL are common indicators for liver’s metabolic capacity [25, 26]. In our study, the increased the levels of liver indicators were observed after parturition, which was in consistency with the report from Cavestany et al., that the partum and postpartum AST activities were higher than prepartum ones [27]. It is worth noting that, the liver indicators in cows with metritis presented even higher values. Similar results have been reported in cows with endometritis [28] and mastitis [29], and suggested that inflammation leads to increased cell membrane permeability and leakage of enzymes into the blood. However, when combined with the higher TG level of the metritis group, we speculated that the continuous utilization of body fat as the source of energy during postpartum NEB status resulted in the TG accumulation within the hepatocytes, impairing liver function [30].

Gluconeogenesis in liver is the main source of blood glucose in ruminants. The increase of glucose at parturition has been well-documented in healthy cows [31]. The decrease of glucose levels a few weeks after parturition may be interpreted as mainly the consequence of the high demand for lactose synthesis [30, 31]. The glucose were lower than the normal range in the metritis group, possibly due to the influence of impaired liver function on gluconeogenesis [30, 32]. This result was generally in agreement with the report from Magnus and Lali [33]. The TG levels in the control group were lower after parturition, this observation was similar to the reports from Mohebbi-Fani et al. [34] and Van den Top et al. [35].

BUN is influenced by many factors, including dietary protein composition and intake, rumen degradability, amino acid catabolism in liver, muscle tissue breakdown, renal excretion, and urea recycling in rumen [26, 36]. CREA represents the mobilization of skeletal muscle, and is excreted via kidney without reabsorption [37]. Both BUN and CREA were within the normal range in all cows, suggesting no obvious kidney injury. The temporary increases of BUN and CREA at 0 d may indicate the mobilization of muscle tissue due to the labor stress [38]. Few study documented the BUN level of dairy cows with metritis. In cows with endometritis, Giuliodori et al. reported no change [39], whereas Senosy et al. found an overall decrease of BUN [40]. In this study, no difference was observed in BUN between the two groups. However, the diseased cows seemed to present a smaller decline of BUN compared with the healthy cows postpartum, which may be interpreted as the reduced metabolic clearance of urea due to the impaired liver function [40]. Similarly, in cows with metritis, the smaller drop of CREA than the healthy cows was observed. This result may suggest a more severe NEB status in cows with metritis.

The onset of lactation places such a large demand on mechanisms of calcium homeostasis that most cows develop some degree of hypocalcemia at calving [30]. The decreased concentrations of Ca and P during and after parturition in both groups corresponded with the statement. We found that the Ca concentrations in cows with metritis was notably lower than the healthy cows, which was in agreement with previous reports [12–14]. It has been shown that the cows develop hypocalcemia at parturition presented higher plasma cortisol concentrations, which exacerbate the immunosuppression status [41]. Hypocalcemia also results in the reduced muscle tone of uterus [42]. Both of these effects account for the increased incidence of postpartum uterine infection.

The pro-inflammatory cytokines, including IL-1, IL-6, IL-8, and TNF-α, have been demonstrated to play an important role in delivery, which is independent of the presence of infection [17, 43]. Similarly, we found increased gene expressions of these cytokines at calving. Ishikawa et al. [44] and Kim et al. [7] reported that the serum levels of IL-6 and TNF-α tended to be lower a few weeks after calving than the prepartum period. Our result showed that the gene expressions of IL-6, IL-8, and TNF-α were lower after parturition than those at − 7 d, which was generally in line with previous reports. The pro-inflammatory cytokines are responsible for inflammatory disorders and the activation of immune cells. The release of these cytokines in the uterus has been confirmed in previous studies [45–47]. Here we detected the elevated gene expressions of these cytokines in cows with metritis after calving, which could be associated with increased tissue cytokine expression. Kasimanickam et al. showed that cows with metritis had higher concentrations of IL-1β, IL-6, and TNF-α compared to normal cows [48]. Together with the increased leucocytes, these results may suggest an altered immune status and a persistent inflammatory response in the peripheral blood.

## Conclusions

Normal fluctuations in the blood leucocytes, biochemistry parameters and the gene expressions of the pro-inflammatory cytokines were observed in healthy dairy cows, which were associated with the parturition and the onset of lactation. In the cows developed metritis, higher WBC, Gran, and Mon, and the elevated gene expressions of IL-1α, IL-1β, IL-6, IL-8, and TNF-α were found after calving, suggesting an altered immune status and a persistent inflammatory response. These cows also presented higher AST activity and TG level, and lower GLU level, which indicated the more intensified NEB status and hepatocyte injury. Lower concentrations of Ca and P in cows with metritis revealed an exacerbated hypocalcemia. In conclusion, our findings suggested that dairy cows with postpartum metritis presented metabolic and immune alterations, as well as impaired liver function.

## Methods

The experiment protocol was approved by the Institutional Animal Care and Use Committee of Yangzhou University. All experimental procedures complied with the Guide for Care and Use of Agricultural Animals of Yangzhou University.

### Animals and experimental design

The study was carried out from September 2016 to November 2016 in a commercial dairy farm of Yangzhou University with 3000 Holstein-Friesian lactating cows. The cows were maintained in free-stall barns and were fed according to the standard guidelines. The total mixed ration included mainly corn silage, alfalfa, soybean meal, distillers dried grains with soluble, cottonseed meal, beet pulp and corn. The cows (662 ± 104 kg) were machine-milked twice daily, with the milk production 31.42 ± 6.75 kg. No difference was found in milk production between these cows.

Before the study, ninety healthy pregnant cows (3 to 5 parities, aged 5 to 7 years) were enrolled 3 weeks before the due date. These cows had normal parturition history and no postpartum disease. They had been checked every other day for 2 weeks by the measurements of rectal temperature (38.0 to 39.3 °C), heart rate (58 to 84 beats per minute), and respiratory rate (26 to 35 breaths per minute), the assessment of body condition score (2.5 to 3.5) and mental state (bright, alert, or responsive), and the visual inspection of the external genitalia (no abnormal vaginal discharge). Seven cows were out of the range of these parameters and were not included in the present experiment. On the day of parturition, all cows routinely receive oxytocin administration. Only cows that had normal unassisted calving were further followed in the study. From 7 to 10 days postpartum, the cows were examined daily by inspection of the external genitalia and measurements of rectal temperature. Metritis was diagnosed if they had dark brown to red color vaginal discharge with malodor, or with increased rectal temperature (≥ 39.5 °C) and other noted clinical signs, such as depression and anorexia. Then the cows were routinely monitored at 17, 24, and 30 days postpartum for assessment of vaginal discharge. The additional transrectal palpation and endometrial cytology were conducted at 24 and 30 days postpartum. The endometrial cytology was carried out as previously described [49]. Briefly, the vulva was cleaned, and cytological samples were collected from cervical mucus by gently rotating the cytobrush while in contact with the endometrium. Strict aseptic procedures were followed and care was taken to avoid trauma. Cytology slides were prepared by rolling the cytobrush on a clean glass microscope slide, followed by fixation and staining using Diff-Quik Stain Kit (Nanjing Jiancheng Bioengineering Institute D030–1, Nanjing, China). Cytological assessment determined the percent neutrophils by counting a minimum of 100 cells at 400× magnification (Nikon Eclipse80i, Tokyo, Japan). After exclusion of the cows with metritis, the rest of the cows were selected as healthy ones if they showed no mucopurulent discharge from vagina at 24 or 30 days postpartum, and less than 18% neutrophils observed in endometrial cytology sample. Based on these examinations, 56 cows were selected in the experiment, with 28 cows in each of the metritis group and the healthy group. During the study period, no treatment was administered. Some of the diseased cows resolved on their own, while others got treatment after the experiment.

### Blood sampling

Whole blood samples were taken at − 7, 0, 7 and 30 d. A total of 10 mL of blood were collected from coccygeal vein into tubes coated with EDTA-Na_2_ for blood routine, and into anticoagulant-free tubes for biochemistry and RNA extraction. Hemolyzed samples were discarded. The blood tubes without anticoagulant were stored for 30 min at 37 °C, followed by centrifugation at 1500×*g* for 15 min. The supernatant (serum) was collected and stored at − 20 °C until biochemical analysis. The deposits were added with 2 mL red blood cell lysis buffer (Beyotime C3702, Shanghai, China), followed by centrifugation at 500×*g* for 5 min at 4 °C to purify the leucocyte deposits, and the supernatant was discarded. This step was repeated until there was no visible red blood cell remnants. Then the deposit was immediately proceeded to the RNA extraction step.

### Blood routine

The blood routine analysis was performed using an automatic hematology analyzer (Mindray BC-2800, Shenzhen, China). The parameters including WBC (10^9^/L), the differential leucocytes (3-differential) counts (10^9^/L) and the percentage (%).

### Biochemical indicators

Serum biochemical parameters were measured by automatic biochemistry analyzer (AU480, Beckdman Counter, USA) using commercial kits (Ningbo Purebio Biotechnology Co., Ltd.). These parameters included: AST (U/L; α-ketoglutarate method), ALT (U/L; α-ketoglutarate method), TBIL (μmol/L; chemical oxidized method), DBIL (μmol /L; chemical oxidized method), glucose (mmol/L; hexokinase method), TG (mmol/L; glycerolphosphate oxidase-PAP method), BUN (mmol/L; urease method), CREA (mmol/L; sarcosine oxidase method), Ca (mmol/L; arsenzao III method), and P (mmol/L; phosphomolybdate method).

### Real-time PCR

Total RNA from the peripheral leukocytes was extracted using Trizol reagent (Invitrogen, CA, USA). The cDNA was synthesized from 1 μg of total RNA using PrimeScript RT Master Mix (Takara Biotechnology, Dalian, China). The β-actin was used as a housekeeping gene. The quantities of mRNA of IL-1α, IL-1β, IL-6, IL-8, and TNF-α relative to β-actin mRNA were determined using the 2^-ΔΔCt^ method by fluorescent quantitative real-time PCR, where ΔCt = Ct _target gene_ – Ct _housekeeping gene_. The sequence of primers (Table 1) were synthesized by Sangon Biotechnology. All the PCR products were purified and sequenced (TsingKe Biotech, Beijing, China) and the sequence results were analyzed using BLAST and compared to GenBank database (https://blast.ncbi.nlm.nih.gov/Blast.cgi).

**Table 1:** Real-time PCR primers and product length

Gene (GeneBank ID)	PCR primer sequences	Product length
β-actin (NM-173979.3)	F:5′-CATCACCATCGGCAATGAGC-3′ R:5′-AGCACCGTGTTGGCGTAGAG-3’	156 bp
IL-1α (NM-174092.1)	F:5’CTAAAGGAGATGCCTGAGACACC-3′ R:5’CTGATTTGAAGTAGTCCATAGAGCC-3’	97 bp
IL-1β (NM-174093.1)	F:5’-TGATGACCCTAAACAGATGAAGAGC-3′ R:5’-CCACGATGACCGACACCACCT-3’	134 bp
IL-6 (EU-276071.1)	F:5’-TGAAAGCAGCAAGGAGACACT-3′ R:5′-TGATTGAACCCAGATTGGAAGC-3’	90 bp
IL-8 (NM-173979.3)	F:5’-TTCCTCAGTAAAGATGCCAATG-3′ R:5′-TGACAACCCTACACCAGACCCA-3’	86 bp
TNF-α (AF-348421.1)	F:5’-GGGCTTTACCTCATCTACTCACAG-3′ R:5′-GATGGCAGACAGGATGTTGACC-3’	132 bp

### Statistical analyses

All data were presented as means ± SD. Data analysis was performed using IBM SPSS Statistics 21.0 (IBM, NY, USA). Normality test was done using the Kolmogorov-Smirnov test, and we used repeated measures ANOVA with Bonferroni post hoc test. A two-tailed *p* value of *<* 0.05 was considered as statistically significant.

## Abbreviations

ALT: Alanine aminotransferase
AST: Aspartate aminotransferase
BUN: Blood urea nitrogen
CREA: Creatinine
DBIL: Direct bilirubin
Gran: Granulocyte count
Gran%: Granulocyte percentage
Lymph: Lymphocyte count
Lymph%: Lymphocyte percentage
Mon: Monocyte count
Mon%: Monocyte percentage
NEB: Negative energy balance
TBIL: Total bilirubin
TG: Triglyceride
